# First report of natural *Wolbachia* infection in wild *Anopheles funestus* population in Senegal

**DOI:** 10.1186/s12936-018-2559-z

**Published:** 2018-11-06

**Authors:** El Hadji Amadou Niang, Hubert Bassene, Patrick Makoundou, Florence Fenollar, Mylène Weill, Oleg Mediannikov

**Affiliations:** 1 Aix Marseille Univ, IRD, AP-HM, MEPHI, IHU-Méditerranée Infection, Marseille, France; 2 Laboratoire d’Ecologie Vectorielle et Parasitaire, Faculté des Sciences et Techniques, Université Cheikh Anta Diop, Dakar, Senegal; 3 VITROME, Campus International, UCAD-IRD, Dakar, Senegal; 4 Aix Marseille Univ, IRD, AP-HM, SSA, VITROME, IHU-Méditerranée Infection, Marseille, France; 5 Institut des Sciences de l’Evolution (ISEM), CNRS-Université de Montpellier-IRD-EPHE, Montpellier, France

**Keywords:** *Wolbachia*, *Plasmodium*, *An. funestus*, *wAnfu*, Malaria, Biological control Senegal

## Abstract

**Background:**

Until very recently, *Anopheles* were considered naturally unable to host *Wolbachia*, an intracellular bacterium regarded as a potential biological control tool. Their detection in field populations of *Anopheles gambiae* sensu lato, suggests that they may also be present in many more anopheline species than previously thought.

**Results:**

Here, is reported the first discovery of natural *Wolbachia* infections in *Anopheles funestus* populations from Senegal, the second main malaria vector in Africa. Molecular phylogeny analysis based on the 16S rRNA gene revealed at least two *Wolbachia* genotypes which were named *wAnfu*-*A* and *wAnfu*-*B*, according to their close relatedness to the A and B supergroups. Furthermore, both *wAnfu* genotypes displayed high proximity with *wAnga* sequences previously described from the *An. gambiae* complex, with only few nucleotide differences. However, the low prevalence of infection, together with the difficulties encountered for detection, whatever method used, highlights the need to develop an effective and sensitive *Wolbachia* screening method dedicated to anopheline.

**Conclusions:**

The discovery of natural *Wolbachia* infection in *An. funestus*, another major malaria vector, may overcome the main limitation of using a *Wolbachia*-based approach to control malaria through population suppression and/or replacement.

## Background

*Wolbachia* are intracellular bacteria found in the cytoplasmic vacuoles of different cells of a wide range of invertebrates, including multiple insect species [1]. The success of *Wolbachia* spp. becoming the most widespread intracellular bacterium, originates from their ability to manipulate the biology of their host to facilitate their maternal transmission to offspring [2, 3]. The most common of these host reproductive manipulation phenotypes, known as cytoplasmic incompatibility (CI), is considered as a potential biological control alternative or complement to traditional vector control measures [4].

Over the past decade, two research groups have reported that *Wolbachia* infection protects against viral RNA infections in *Drosophila melanogaster* [5, 6]. Subsequently, the successful trans-infection of *Aedes aegypti* with a *Drosophila Wolbachia* strain has opened a new era for environmental-friendly control strategies of main mosquitoes-borne diseases using *Wolbachia*-based strategies [7]. Moreover, successful releases of *Wolbachia*-infected *Aedes aegypti* in Australia has provided experimental validation of previous theoretical models of *Wolbachia* population dynamics and demonstrated the viability of *Wolbachia*-based vector control strategies [8]. For decades, anopheline mosquitoes that transmit human malaria have been considered resistant or less susceptible to *Wolbachia* infections due to the failure to detect native *Wolbachia* infection in 38 species of anopheles [9, 10], and the impossibility to obtain stable *Wolbachia* trans-infected anopheline lines [11, 12]. Although, *Wolbachia* trans-infections have been attempted with success in both *Anopheles gambiae* [11, 13] and *Anopheles stephensi* [14], respectively major vectors of human malaria in Africa and the Middle East, and South Asia [15, 16], their impact on *Plasmodium* development were not always conclusive. The dogma of the absence of native *Wolbachia* infection in anopheline mosquito has recently changed with the first report of *Wolbachia* in *Anopheles gambiae* sensu lato (s.l.) from Burkina Faso [17, 18]; and more recently from Mali [10].

Recent advances in the molecular biology area, including sequencing of 16S rRNA, have revolutionized the characterization of several fastidious microorganisms. This is particularly true for intracellular bacteria of the members of the *Wolbachia* genus [19]. Indeed, phylogenetic analyses of the 16S rRNA gene have revealed that *Wolbachia pipientis*, the *nomen* species of the genus, forms a monophyletic clade within the *Alphaproteobacteria* class, closely related to the *Anaplasma*, *Ehrlichia* and *Neorickettsia* genera of the *Anaplasmataceae* family [20]. Further phylogenetic analysis based on the 16S rRNA of newly discovered anopheline *Wolbachia* strains revealed that they belong to a new phylogenetic group called *wAnga* related to, but distinct from *Wolbachia* infecting other arthropods [9, 10, 17]. Baldini et al. [17], explain previous failure of detecting *Wolbachia* in anopheline mosquitoes as possibly due to the lack of sufficiently sensitive detection systems and developed a nested amplification method. In addition, the new *wAnga* genotypes display a low degree of sequence conservation compared to previously described genotypes isolated in other insects leading to unsuccessful amplification of several genes commonly used for Multi-Locus Sequence Typing (MLST) *Wolbachia* universal genotyping tool [17]. These discoveries suggest that more anopheline species, including others major malaria vectors, may also be infected by *Wollbachia*.

In this study, is reported the first discovery of natural *Wolbachia* infections in *Anopheles funestus* in Senegal, the second main malaria vector in Africa.

## Methods

### Study area and mosquito collection

A total of 247 adult females of *An. funestus* collected during the raining season of the year 2014 were screened. Samples were randomly selected from an existing mosquito collection from the longitudinal cohort study conducted since 1990 in the village of Dielmo [Senegal]. Detail of the study village and mosquito collection methods are described elsewhere [21, 22]. All specimens were identified morphologically as *An. funestus* using the taxonomic key of Gillies and Meillon [23] prior to molecular detection and genotyping of *Wolbachia* spp.

### Molecular detection and phylogenetic genotyping of *Wolbachia*

Genomic DNA was extracted from the abdomen of individual mosquito using the Biorobot EZ1 System with the EZ1 DNA tissue kit [Qiagen, Court a boeuf, France] following the manufacturer’s instructions. Individual mosquitoes were screened as described in Shaw et al. [18] using both the standard [W16S-Spec] [24] and the nested PCR [W16S-Nested] [17] protocols. A third qPCR assay [W16S-qPCR] recently developed [10] was used to confirm *Wolbachia* infection in *An. funestus*.

The nested-16S rDNA *Wolbachia* primers were used to generate a 340–412-bp fragment according to Baldini et al. [17]. The PCR products of all *Wolbachia*-positive samples were purified by filtration using NucleoFast^®^ 96 PCR DNA purification plate then amplified using the BigDye™ Terminator v3.1 Cycle Sequencing Kit [Applied Biosystems, Foster City, CA]. The BigDye PCR products were purified on the Sephadex G-50 Superfine gel filtration resin prior the sequencing on the ABI Prism 3130XL.

### Phylogenetic analysis of *Wolbachia*

Nucleotide sequences were edited using ChromasPro 2.0.0, then aligned against close reference sequences of *wMel* [LC108848] and *wAlb,* [AM999887], respective representatives of the A and B supergroups; and three *wAnga* genotypes, previously described in field populations of *An. gambiae* s.l. from Burkina [KJ728749 and KJ728744] and from Mali [MF944114]. All the reference sequences were retrieved from the GenBank database and the alignment was performed using the ClustalW application within Bioedit v.7.2.5. [25]. Nucleotides conservation between the *An. funestus Wolbachia* sequences comparatively to reference sequences was visualized on CLC Sequence Viewer 7 (CLC Bio Qiagen, Aarhus, Denmark).

The maximum likelihood phylogenetic tree was inferred on Topali v2.5 [26], based on the Kimura three-substitution-type substitution model [27].

## Results

### *Wolbachia* infection in wild *Anopheles funestus* populations from Senegal

*Wolbachia* DNA was detected in three specimens out of 247 females of *An. funestus* tested, which corresponds to a frequency of infection of 1.21%. This is the first report of *Wolbachia* infection among natural population of *An. funestus* in Senegal. Despite several attempt, the quantitative PCR developed by Gomez et al. [10] failed to amplify the three positive samples amplified with the nested PCR.

### Phylogenetic analysis of *Wolbachia* infecting *Anopheles funestus*

**Fig. 1:**
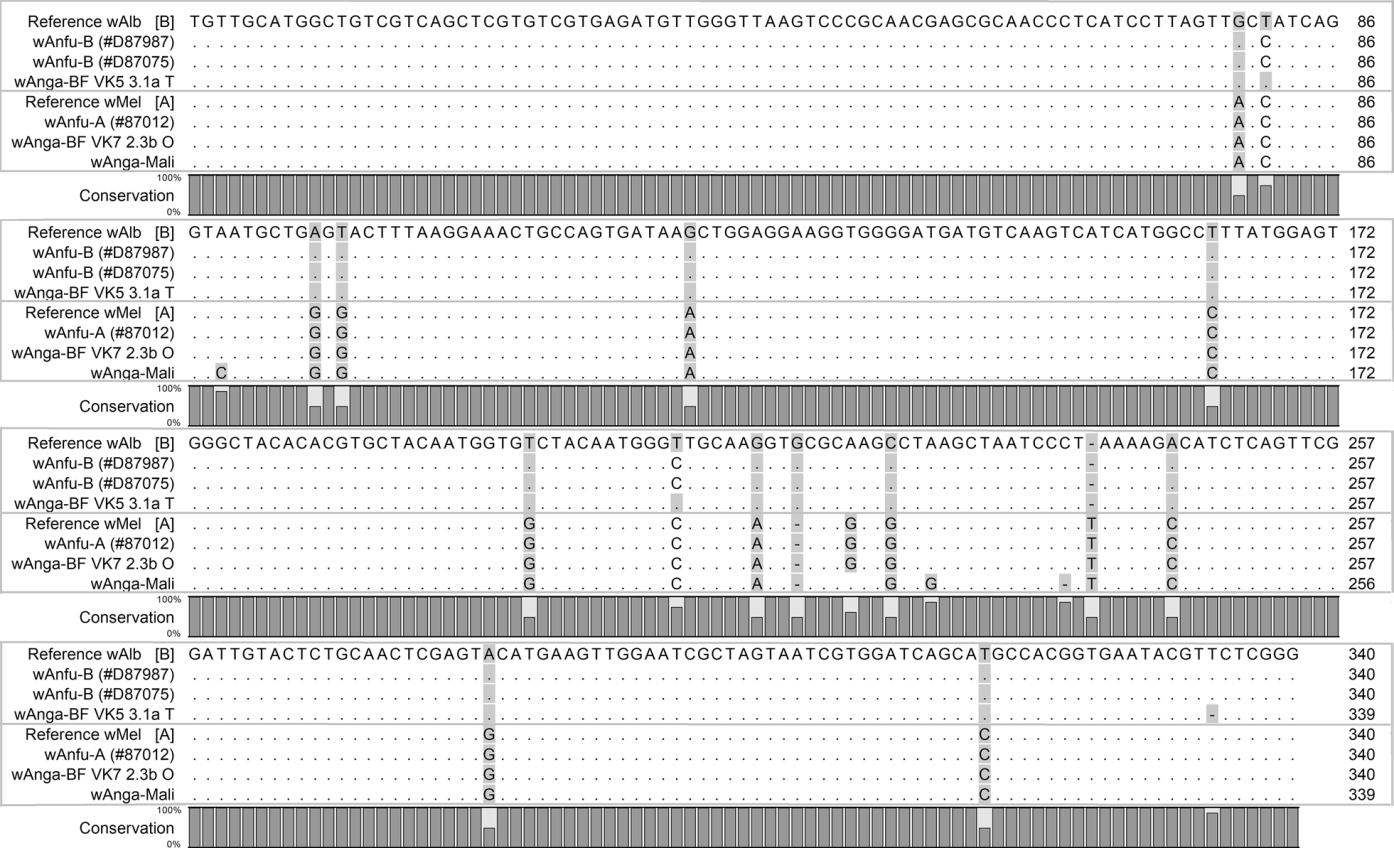
*wAnfu* sequences alignment against reference sequences. The sequences of the three *Wolbachia*-positive *An. funestus* samples were aligned against close reference sequences, representative of the A supergroup (wMel; [LC108848]), the B supergroup (wAlb; [AM999887]) and the *wAnga* group from Burkina Faso (*wAnga* VF7 2.3b O [KJ728749] and *wAnga* VF5 3.1a T [KJ728744]) and from Mali (*wAnga*-Mali [MF944114])

**Fig. 2:**
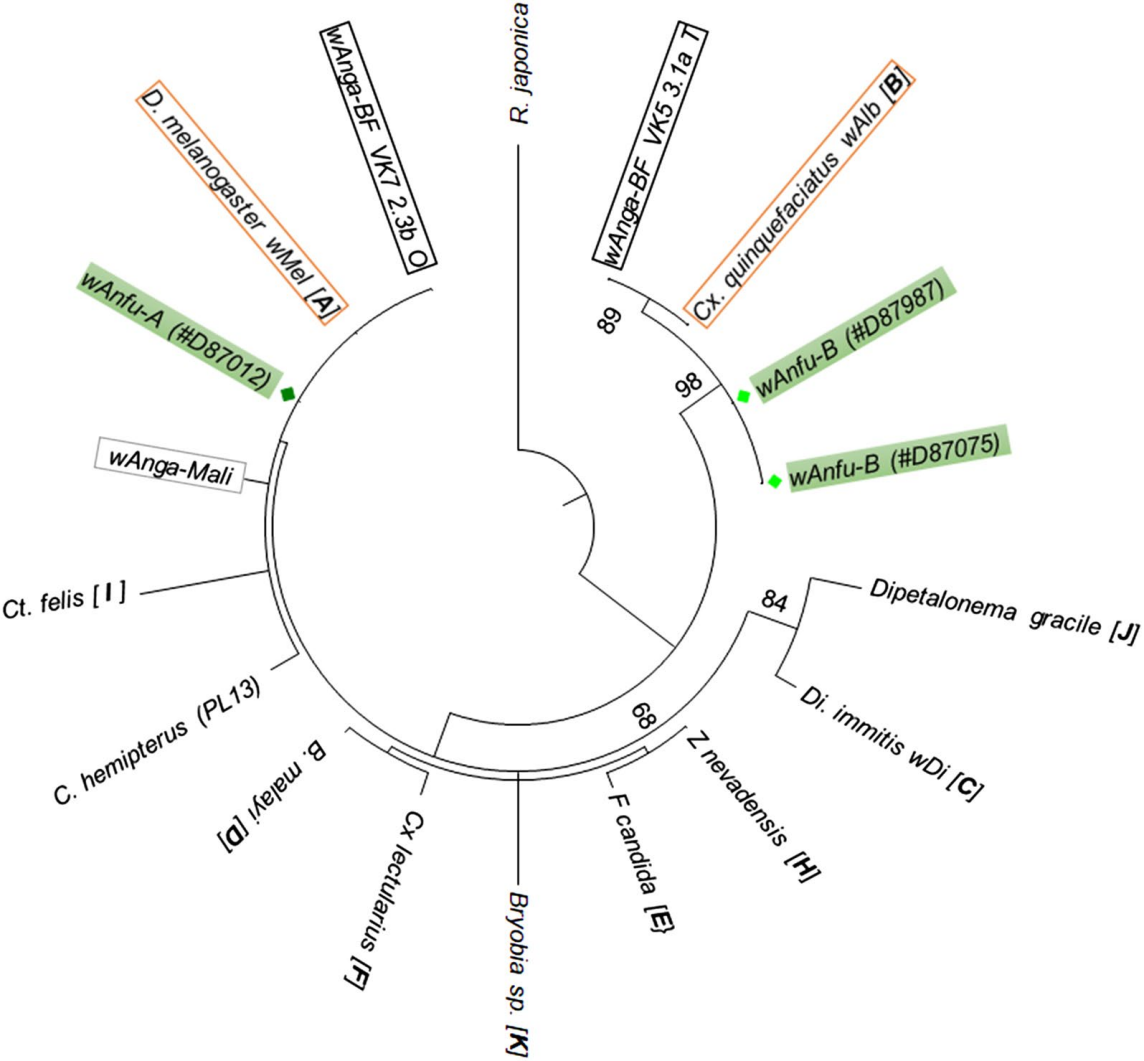
Molecular Phylogenetic analysis by the Maximum Likelihood method. The evolutionary history based on the 340 bp sequence of the partial *Wolbachia* 16S rRNA gene was inferred using the Maximum Likelihood method based on the Kimura three-substitution-type model [27]. *Wolbachia* genotypes of *An. funestus* are highlighted by green shaded boxes. The two red boxes show the A and B supergroups reference sequences. The *wAnga* genotypes from Burkina Faso are highlighted with black boxes and *wAnga*-Mali is illustrated by a grey box. The different *Wolbachia* supergroups are mentioned in brackets after each reference genotype name

Phylogenetic analysis of the16S rRNA gene revealed that the *An. funestus* samples from Senegal infected with at least two *Wolbachia* genotypes which cluster with the A or B clades (Figs. 1, 2), and were named *wAnfu*-A and *wAnfu*-B. Further sequences comparisons showed the identity of *wAnfu*-A to *wMel* and *wAnga*-BF VK7; and the close relatedness of *wAnfu*-B sequence to *wAlb* and *wAnga*-BF VK5 (Fig. 2). Noteworthy, despite their proximity, slight levels of divergence were found between the *wAnfu* genotypes of *An. funestus* and *wAnga* strains previously found in the *An. gambiae* complex. Furthermore, mutations differentiating the two *wAnfu* variants suggest further diversification within *Wolbachia* strain infecting *An. funestus*.

## Discussion

This is the first report of *Wolbachia* infection among natural population of *An. funestus* in Senegal, the second main malaria vector in the African continent [15]. This extends the presence of *Wolbachia* to another anopheline species since its first discovery in field populations of *An. gambiae*, *Anopheles coluzzii* and *Anopheles arabiensis* from Burkina Faso [17, 18] and in *An. gambiae* and *An. coluzzii* from Mali [10]. However, the prevalence of *Wolbachia* in *An. funestus* is lower compared to the previous reports, with 46% (275/602) in 2014 in Burkina Faso and varying spatially and temporally in Mali with a minimal infection rate of 45% in Dangassa in 2015 reaching 95% (38/40) in Kenieroba in 2016. Such differences in prevalence may be explained by lower infection rates in *An. funestus* compared to *An. gambiae* species [10, 17, 18] due to biological, immunological or any other factors. Another hypothesis could be a weaker infection density in *An. funestus* [quantity of *Wolbachia* per cell] that could prevent its correct detection. The failure of the qPCR assay developed by Gomez et al. [10] to amplify the three positive samples, confirmed independently in two laboratories (in Marseille and Montpellier), suggests such an explanation, since the sequences of the primers designed for this qPCR are conserved in the *Wolbachia* of *An. funestus*. Thus, the *wAnfu* prevalence detected in Senegal is minimal and could be higher with a more efficient detection system. Moreover, a certain level of divergence between *wAnfu* and *wAnga* sequences previously found in the *An. gambiae* complex [10, 17, 18] could also prevent efficient amplification and thus detection. Indeed, Baldini et al. [17], also attributed the previous failure to detect *Wolbachia* infection in wild anopheline populations by non-optimal detection tools, due to the genetic divergence of the new *wAnga* strain from *Wolbachia* found in other insects. Finally, because of the low prevalence (1.2%) of *Wolbachia* infection in *An. funestus*, probably due to the low infection level that can lead to qPCR failures, and in the absence of vertical transmission history, we cannot exclude the possibility of an occasional unstable infection.

Phylogenetic analysis showed that natural populations of Senegalese *An. funestus* harbour at least two distinct *Wolbachia* genotypes; clustering respectively with the clade A and B commonly encountered in the *Arthropoda* phylum [3]. The new genotypes were therefore named *wAnfu*-*A* and *wAnfu*-*B*, according to their respective relatedness to the A and B supergroups. Indeed, further analyses revealed the similarity of *wAnfu*-A to *wMel* and *wAnga*-BF VK7; while *wAnfu*-B was closer to *wAlb* and *wAnga*-BF VK5. However, multi-sequence alignment revealed that despite their proximity, the *An. funestus Wolbachia* genotypes were slightly different from the previously described *wAnga* infecting several species of the *An. gambiae* complex [17, 18].

Moreover, the appearance of some mutations also favours diversification between *Wolbachia* in *An. funestus*, with at least two variants. Given the suspected further divergence between anopheline *Wolbachia* groups, some genotypes may have been missed. It is, therefore, critical to vary detection methods targeting more genes. A classic approach to characterize *Wolbachia* genotypes and clades is based on the MLST system using internal fragments of five ubiquitous genes (*gatB, coxA, hcpA, fbpA,* and *ftsZ*) [28]. However, as shown by Baldini et al. [17], their newly identified *wAnga* genotype was highly divergent from groups isolated in other insects, leading potentially to the presence of null alleles if mutations have occurred in the region targeted to design the standard primers of the MLST universal genotyping tool. This seems to be corroborated by Gomes et al. [10], who failed to successfully amplify the *gatB* and *ftsZ* genes. There is, therefore, an urgent need to develop an optimal screening method, but also a specific MLST system for anopheline *Wolbachia*. A critical and challenging step for this would be the isolation and whole genome sequencing of, as much as possible, *Wolbachia* genotypes infecting anopheline mosquitoes to come-up with a specific MLST system and potentially a more efficient screening system. Then, more data on *Wolbachia* prevalence will be required to further assess their potential role in impeding the development of *Plasmodium* or any other parasites.

This is the first report of the presence of *Wolbachia* spp. in *An. funestus* from Senegal. Stable natural *Wolbachia* carriage among main malaria vectors may overcome the main limitation of using a *Wolbachia*-based approach to control malaria through population suppression and/or replacement. However, further studies are needed to better characterize the diverse *Wolbachia* groups infecting anopheline mosquitoes prior they could be efficiently used as control tool.

## Abbreviations

DNA: deoxyribonucleic acid
MLST: multilocus sequence typing
PCR: polymerase chain reaction
qPCR: quantitative polymerase chain reaction
RNA: ribonucleic acid
